# The role of the GABAergic cells of the median raphe region in reinforcement-based learning

**DOI:** 10.1038/s41598-024-51743-y

**Published:** 2024-01-12

**Authors:** Tiago Chaves, Bibiána Török, Csilla Fazekas, Pedro Correia, Peter Karailiev, Henrieta Oravcova, Eszter Sipos, László Biró, József Haller, Daniela Jezova, Dóra Zelena

**Affiliations:** 1 Institute of Physiology, Medical School, Centre for Neuroscience, Szentágothai Research Centre, University of Pécs, 7624 Pecs, Hungary; 2 Laboratory of Behavioural and Stress Studies, Institute of Experimental Medicine, Budapest, Hungary; 3 János Szentágothai Doctoral School of Neurosciences, Semmelweis University, Budapest, Hungary; 4 Biomedical Research Center, Institute of Experimental Endocrinology, Slovak Academy of Sciences, Bratislava, Slovakia; 5 Department of Pharmacology and Toxicology, Faculty of Pharmacy, Comenius University Bratislava, Bratislava, Slovakia; 6 Ludovika University of Public Service, Budapest, Hungary

**Keywords:** Cognitive neuroscience, Learning and memory

## Abstract

Learning and memory are important in everyday life as well as in pathological conditions. The median raphe region (MRR) contributes to memory formation; however, its precise role and the neurotransmitters involved have yet to be elucidated. To address this issue, we stimulated the MRR neurons of mice by chemogenetic technique and studied them in the operant conditioning and active avoidance tests. The virus carrier infected a variety of neuron types including both GABAergic and glutamatergic ones. Behavior was not influenced by stimulation. We hypothesize that the lack of effect was due to opposing effects exerted via GABAergic and glutamatergic neurons. Therefore, next we used VGAT-Cre mice that allowed the specific manipulation of MRR-GABAergic neurons. The stimulation did not affect behavior in the learning phase of the operant conditioning task, but increased reward preference and total responses when operant contingencies were reversed. The enhanced responsiveness might be a proclivity to impulsive behavior. Stimulation facilitated learning in the active avoidance test but did not affect reversal learning in this paradigm. Our findings suggest that MRR-GABAergic neurons are involved in both learning and reversal learning, but the type of learning that is affected depends on the task.

## Introduction

Memory and learning are fundamental cognitive processes, in which both the stimulatory glutamate^1,2^ and the inhibitory gamma-aminobutyric acid (GABA)^3,4^ neurotransmitters play a pivotal role. The role of glutamate is somewhat better known^5^, but it has also been shown that systemic post-training injections of GABAergic compounds (such as the antagonist picrotoxin or the agonist muscimol) can also modulate memory^6^. Other studies revealed the effect of bicuculline (another antagonist of GABA) in memory facilitation during post-training administration into the hippocampus, entorhinal cortex and parietal cortex of rats^7^, and in memory consolidation in an invertebrate model^8^. Dysregulation of GABAergic activity in the prefrontal cortex of elderly rats negatively influenced their working memory performance^9^. Moreover, in humans, low GABA levels of the prefrontal cortex went along with worser working memory after an increased workload^10^. It is important to note that published studies have suggested a general effect, but a complete understanding of the contribution of different brain areas to these processes is still evolving.

We focused on the median raphe region (MRR), which is located in the midbrain, and is implicated in the regulation of several cognitive and behavioral functions, among others in fear behavior^11^, memory consolidation^12^ and reward-related behavior^13^. Although the MRR is widely known as a serotoninergic area, there is growing evidence pointing to the presence of non-serotoninergic neurons^14,15^. In fact, it has been quantified that the majority of the neurons in the MRR are GABAergic^16^. However, it is yet to be elucidate the role of this neuron population in the MRR.

Chemogenetic technique (designer receptors exclusively activated by designer drugs (DREADD) alongside with its artificial ligand, clozapine-N-oxide (CNO)) allows accurate manipulation of desired neurons on a well-defined brain area. Thus, it seems to be a suitable technique for testing the present hypothesis that the stimulation or inhibition of GABAergic neurons of the MRR influences learning and memory formation. Initially, we manipulated the whole MRR. A significant portion of the infected cells were found to be GABAergic, however, glutamatergic cells were labeled similarly. Thus, the simultaneous manipulation of stimulatory and inhibitory neurons might have counteracted each other. Therefore, as the next step, we used a mice line containing Cre recombinase enzyme under the vesicular GABA transporter (VGAT) promoter to investigate possible effects that MRR GABAergic neurons might have. With regard to behavioral measures, we focused on operant conditioning and active avoidance tests (consecutively) due to their sensitivity to cognitive changes and the fact that among others they assess behavioral functions known to be under the control of the MRR^11–13^. These two reinforcement-based cognitive tests are different in nature, one is a reward driven (operant conditioning), while the other is a punishment avoiding (active avoidance) paradigm, what rationalizes the use of both.

## Results

### Experiment 1

**Figure 1:**
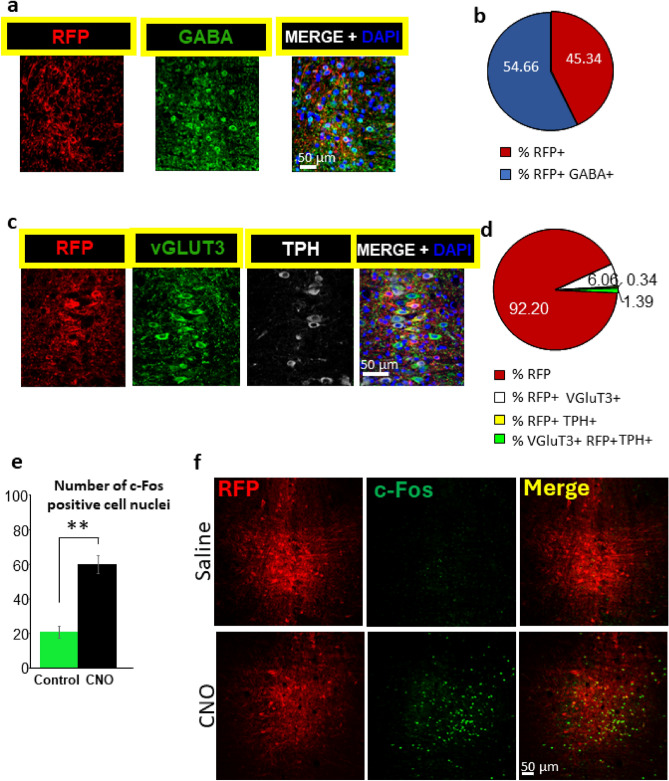
Colocalization of GABAergic and viral infection (RFP) markers. (**a**) Representative picture of the MRR cells infected by AAV containing RFP (A594) as a reporter protein. Majority of the neurons are GABA positive (A488) as well (×40 magnification) (**b**) Almost half of all AAV infected (RFP positive) cells were also GABAergic. (**c**) Representative picture of the RFP positive (A594), vGLUT3 positive (A488) and TPH positive (A633) neurons of the MRR (×40 magnification). (**d**) Ratio (%) of different neuron types based on their expressed neurotransmitter markers in the MRR. Only minority of the cells were serotonergic (TPH positive) and/or glutamatergic (VGluT3 positive) compared to all AAV infected (RFP positive) cells. (**e**) Representative picture of neuronal activation of marked cells studied by RFP and c-Fos colocalization. (**f**) The excitation of the whole MRR in C57BL/6J mice via excitatory DREADD resulted in a marked increase of c-Fos positive neuron nuclei compared to saline injected control animals. Data are expressed as mean ± SEM. Data were compared to each other with t-test. **p < 0.01 vs control. AAV: adenoassociated viral vector; CNO: clozapine-N-oxide; DREADD: designer receptor exclusively activated by designer drugs; MRR: median raphe region; RFP: red fluorescent protein; TPH: tryptophan hydroxylase VGluT3: vesicular glutamate transporter type 3.

The immunohistochemical analyses revealed 47.9% of cells infected by the virus carrier were stained neither for the GABAergic marker nor for the vesicular glutamate transporter 3 (VGluT3) or tryptophan hydroxylase (TPH). The majority of stained neurons were GABAergic as shown by the co-localization of anti-RFP (red fluorescent protein) and GABA labeling (43.1%) (Fig. 1a,b). Much less prevalent were the serotonergic (TPH-expressing) (1.89%) and glutamatergic (VGluT3-expressing, highly abundant in MRR^16^) cells (8.31%) (Fig. 1c,d). As expected, intraperitoneal CNO injection increased the share of c-Fos positive cells (Fig. 1e,f).

#### Operant conditioning

**Figure 2:**
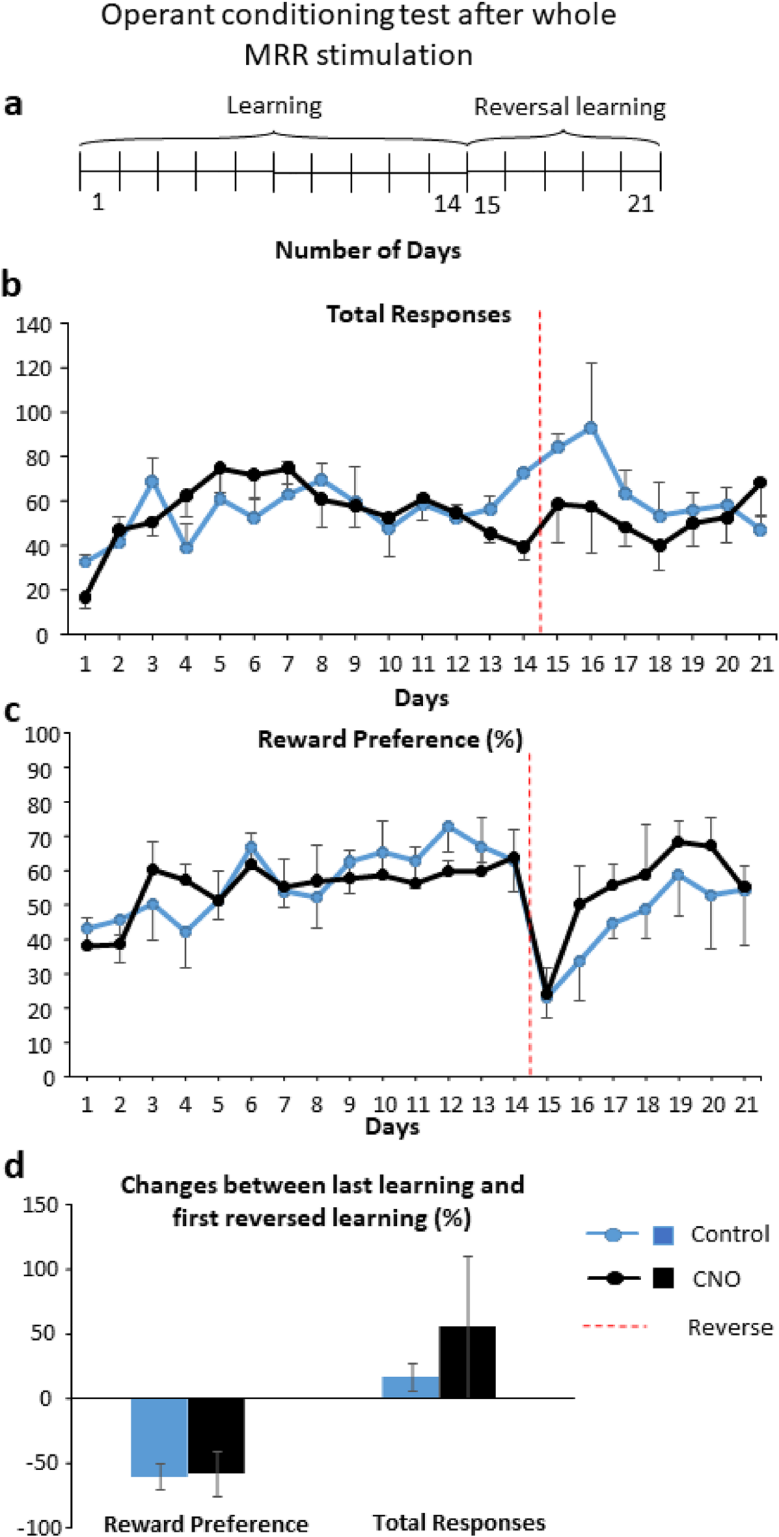
Operant conditioning test after whole MRR stimulation (Experiment 1). (**a**) Schematic timeline of the operant conditioning test. The learning phase consisted of 14 days while the reversal learning phase lasted for 7 days, each with 30-min-long training sessions per day. (**b**) Total numbers of responses (correct + incorrect) during learning phase increased across the days, without any difference between the groups. During reversal learning phase (indicated by the red dashed line) the total number of responses was marginally lower for the stimulated group. (**c**) Reward preference (percentage of correct nose pokes vs total nose pokes) did not differ throughout the whole experiment and did not reach the random chance 50%. (**d**) Percentage of change between the first day of reversal learning and last day of learning phase for total responses and reward preference, respectively. There were no differences between the groups. Data are expressed as mean ± SEM. Data were compared to each other with repeated-measures ANOVA (total responses, reward preference), single sample t-test (vs random chance 50%) and t-test (change between last and first). CNO: clozapine-N-oxide, MRR: median raphe region.

Total number of responses increased across the days of learning, indicating that the animals learned the paradigm (significant time effect; see Table 1; Fig. 2). There was no significant difference between the two groups (control and MRR stimulation) during the learning phase (no treatment effect or interaction with time). Moreover, no consistent difference was found in the single sample t-test when analyzing the preference of baited nose hole in comparison to the chance level of 50% (p > 0.05, Suppl. Table 1).

**Table 1:** Statistical details for the whole median raphe stimulation (Experiment 1) analyzed by repeated measures ANOVA.

Experiment		Parameters	Effect	Degree of freedom	F	p
Operant conditioning	Learning	Total responses	Treatment	1,13	1.620	0.225
			Time	13,169	4.657	0.000
			Time × treatment	13,169	0.399	0.968
		Reward preference	Treatment	1,13	0.022	0.882
			Time	13,169	2.591	0.002
			Time × treatment	13,169	0.666	0.793
	Reversal learning	Total responses	Treatment	1,12	2.614	0.131
			Time	6,72	3.300	0.006
			Time × treatment	6,72	2.117	0.061
		Reward preference	Treatment	1,12	0.144	0.710
			Time	6,72	11.431	0.000
			Time × treatment	6,72	0.418	0.864
	Learning + reversal learning	Total responses	Treatment	1,12	3.521	0.085
			Time	20,240	4.080	0.000
			Time × treatment	20,240	0.960	0.511
		Reward preference	Treatment	1,12	0.133	0.722
			Time	20,240	4.503	0.000
			Time × treatment	20,240	0.494	0.967
Active avoidance	Learning	N# of EDST	Treatment	1,7	0.007	0.933
			Time	4,28	16.205	0.000
			Time × treatment	4,28	1.954	0.115
		N# of EDFS	Treatment	1,7	0.250	0.625
			Time	4,28	8.000	0.000
			Time × treatment	4,28	0.388	0.815
		N# of ESFL	Treatment	1,7	0.386	0.544
			Time	4,28	0.810	0.524
			Time × treatment	4,28	0.759	0.556
		Average latency to escape	Treatment	1,12	0.023	0.883
			Time	4,28	13.190	0.000
			Time × Treatment	4,28	0.634	0.641
	Reversal learning	N# of EDST	Treatment	1,7	1.053	0.323
			Time	4,28	16.419	0.000
			Time × treatment	4,28	0.709	0.589
		N# of EDFS	Treatment	1,7	1.862	0.195
			Time	4,28	0.923	0.457
			Time × treatment	4,28	1.478	0.222
		N# of ESFL	Treatment	1,7	1.372	0.262
			Time	4,28	7.288	0.000
			Time × treatment	4,28	2.270	0.074
	Learning + reversal learning	N# of EDST	Treatment	1,13	0.000	0.982
			Time	9,117	22.157	0.000
			Time × treatment	9,117	0.838	0.582
		N# of EDFS	Treatment	1,13	1.474	0.246
			Time	9,117	32.055	0.000
			Time × treatment	9,117	0.335	0.962
		N# of ESFL	Treatment	1,13	1.478	0.246
			Time	9,117	111.291	0.000
			Time × treatment	9,117	0.933	0.499

EDST: escape during stimulus; EDFS: escape during footshock; ESFL: escape failure.

On day 15, at the beginning of the reversal learning phase, the successful responses dropped for both groups, as expected (Fig. 2). On days 15–21 the performance of the MRR-stimulated mice measured by total responses was marginally worse than that of the control mice (Table 1. treatment x time interaction: p = 0.061), however, there were no significant differences between the two groups for the reward preference. Additionally, there was no significant difference in reward preference (t(13) = 0.651, p = 0.526) and total responses (t(13) = 1.221, p = 0.244) between the groups during the last day of learning and the first day of reversal learning (i.e. during "switch", calculated as Day15/Day14*100) (Fig. 2d). Moreover, for both parameters (reward preference and total responses) only the time effect was significant for the whole 21 days observation period either, with significant change during the first day of reversal learning phase compared to other days during Bonferroni posthoc analysis (Table 1).

#### Active avoidance

**Figure 3:**
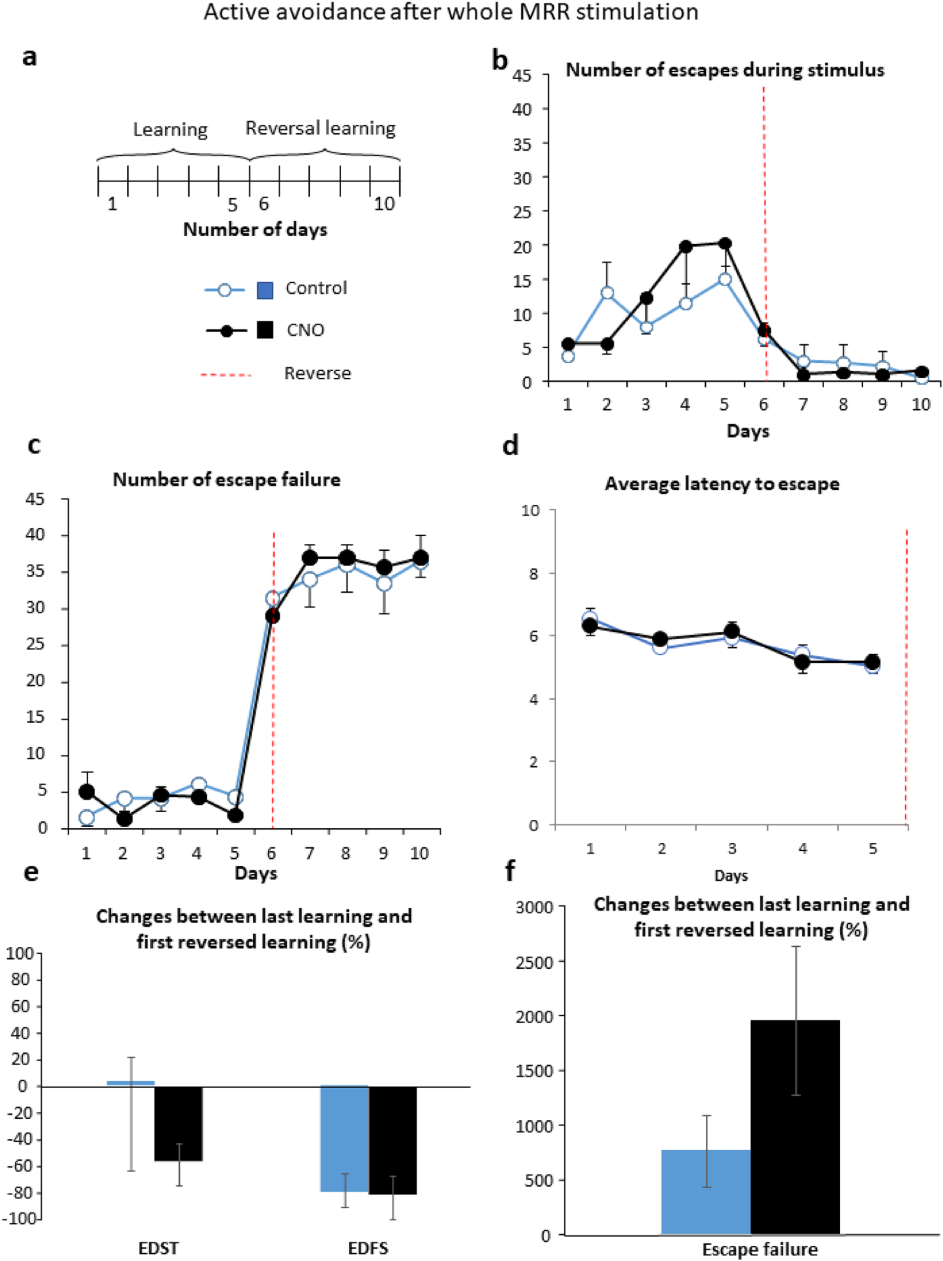
Active avoidance after whole MRR stimulation (Experiment 1). (**a**) Schematic timeline of the active avoidance test. The experiment lasted for a total of 10 days, 5 days of learning and 5 days of reversal learning phase. Each day there were 40 × 30 s long trials, with 1 min habituation before the start and 5 s intertrial interval. Learning was helped with sound and light cues. (**b**) During learning, the number of successful escapes increased without differences between the groups. Similarly, successful learning during reversal learning phase (indicated by red dashed line) was indicated by the rapid drop in the number of escapes, without an effect of treatment. (**c**) The number of escapes failures increased during reversal learning, without differences between the groups. (**d**) Average escape latency during learning as a possible measure of impulsivity. (**e**) Percentage of change between last day of learning phase and first day of reversal learning for EDST and EDFS did not differ between the groups. (**f**) Percentage of change between last day of learning phase and first day of reversal learning for escape failures did not differ between the groups. Data are expressed as mean ± SEM. Data were compared to each other with repeated-measures ANOVA (EDST, EDFS, escape failures) and t-test (change between last and first). CNO: clozapine-N-oxide; EDFS: escapes during footshock; EDST: escapes during stimulus; MRR: median raphe region.

During learning (days 1–5, Fig. 3a, Table 1) the number of escapes during stimulus (EDST) increased in both groups suggesting successful learning (Fig. 3b). Complementary to this, escape during footshock (EDFS) decreased gradually (data not shown). The escape failure was rather low and did not improve significantly during learning (Fig. 3c). There were no differences between the groups in the above mentioned parameters as well as in the impulsivity marker average escape latencies (Fig. 3d).

When the animals had to learn not to escape (during the reversal learning phase; days 6–10), there was a significant drop in their escape (EDST Fig. 3b), which remained low during the subsequent days, suggesting a rather fast adaptation. No significant difference was observed between the groups (Fig. 3). Moreover, the number of escape failures (in fact during this phase it was a correct behavioral answer) increased in both groups throughout the days, with a marginal group x time interaction effect (p = 0.07; Table 1). In addition, when we expressed the changes between the last day of learning and the first day of reversal learning there was no significant difference between the groups (Fig. 3e,f).

### Experiment 2

#### Operant conditioning

**Figure 4:**
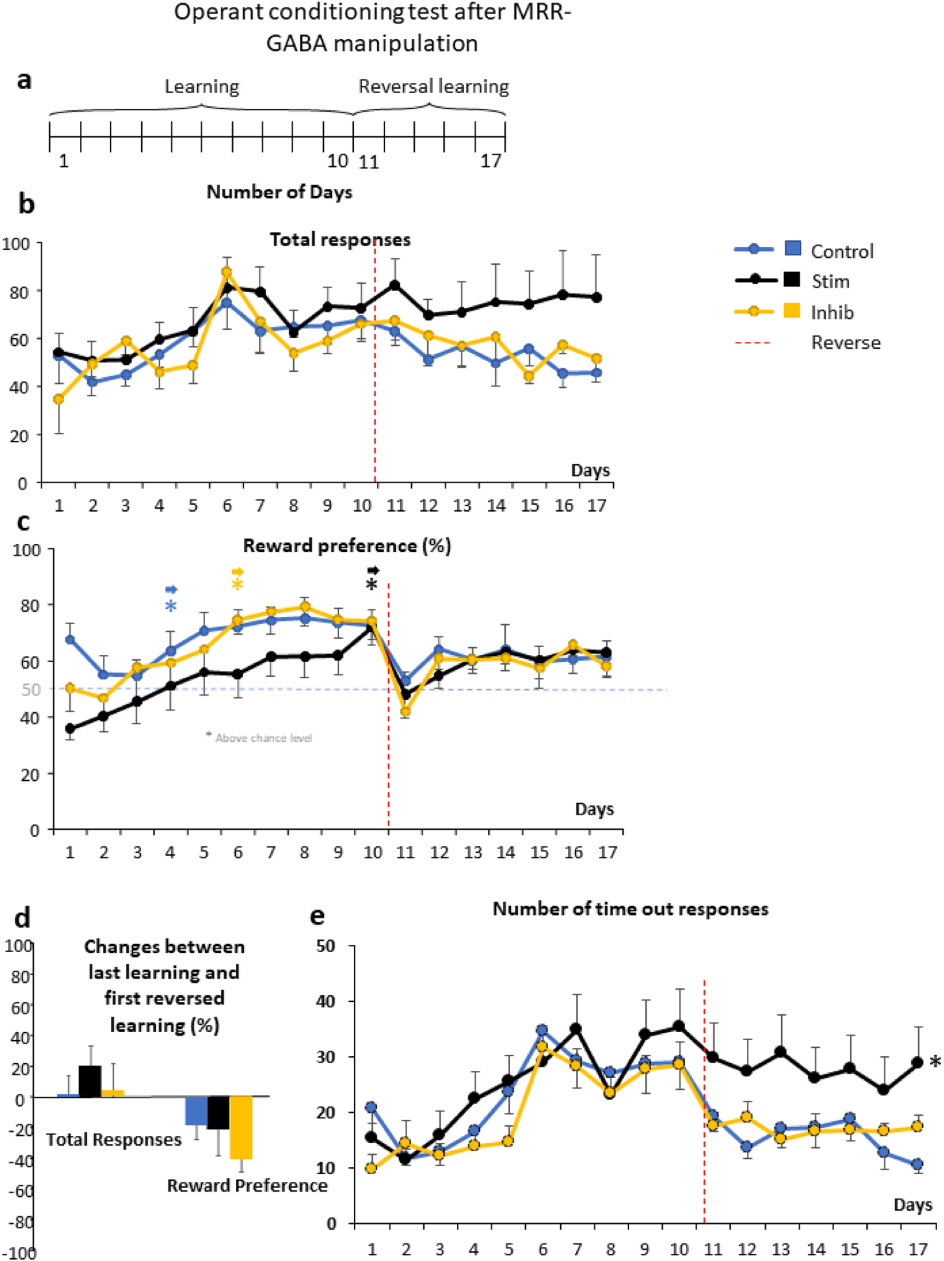
Operant conditioning test after manipulation of the GABAergic cells of the median raphe region (Experiment 2). (**a**) Schematic timeline of the operant conditioning test carried out similarly as during Experiment 1. In this case, the learning phase lasted for 10 days, while the reversal learning phase lasted for 7 days. (**b**) The number of total responses (correct + incorrect) increased throughout the days, without any effect of treatment. However, during reversal learning (indicated by red dashed lines) the stimulatory grouped had higher number of total nose pokes compared to the other two groups. (**c**) Reward preference (percentage of correct nose pokes vs total nose pokes) did not differ between the groups, but the control and inhibitory group reached random chance 50% (indicated by blue dashed lines) sooner (4th and 5th day, respectively) than the stimulatory group (10th day). During reversal learning phase (indicated by red dashed lines), only the stimulatory group kept their performance above random chance 50% (indicated by blue dashed lines). (**d**) Percentage of changes between last day of learning phase and first day of reversal learning for total number of responses and reward preference showed no differences between the groups. (**e**) Number of timeout responses as possible sign of impulsivity. Data are expressed as mean ± SEM. Data were compared to each other with repeated-measures ANOVA (total responses, reward preference) and single sample t-test (vs random chance 50%). *p < 0.01 vs random chance 50%. ^#^p < 0.01 main treatment effect during Bonferroni posthoc comparison, stimulatory group vs. control as well as inhibitory groups. Inhib: inhibitory receptor sequence containing virus vector, MRR: median raphe region, Stim: stimulatory receptor sequence containing virus vector.

In chemogenetically MRR-GABA manipulated mice the successful learning of the paradigm was reflected by a gradual increase of the total number of responses across the days of learning (Fig. 4b, Table 2).

**Table 2:** Statistical details for manipulation of the GABAergic cells of the median raphe region (Experiment 2) analyzed by repeated measures ANOVA.

Experiment		Parameters	Effect	Degree of freedom	F	p
Operant conditioning	Learning	Total responses	Treatment	2,35	1.410	0.257
			Time	9,315	13.772	**0.000**
			Time × treatment	18,315	1.094	0.356
		Reward preference	Treatment	2,35	1.163	0.324
			Time	9,315	16.807	**0.000**
			Time × treatment	18,315	0.909	0.567
		Timeout response	Treatment	2,35	0.539	0.588
			Time	9,315	16.883	**0.000**
			Time × treatment	18,315	1.146	0.307
	Reversal learning	Total responses	Treatment	2,35	4.031	**0.027**
			Time	6,210	2.941	**0.009**
			Time × treatment	12,210	0.567	0.866
		Reward preference	Treatment	2,35	3.721	**0.035**
			Time	6,210	7.652	**0.000**
			Time × treatment	12,210	0.932	0.515
		Timeout response	Treatment	2,35	3.822	**0.031**
			Time	6,210	0.461	0.837
			Time × treatment	12,210	0.512	0.906
	Learning + reversal learning	Total responses	Treatment	2,32	2.566	*0.092*
			Time	16,512	9.561	**0.000**
			Time × treatment	32,512	0.974	0.511
		Reward preference	Treatment	2,35	1.153	0.328
			Time	16,560	9.748	**0.000**
			Time × treatment	32,560	1.584	**0.024**
		Timeout response	Treatment	2,35	1.753	0.188
			Time	16,560	9.174	**0.000**
			Time × treatment	32,560	1.417	*0.066*
Active avoidance	Learning	N# of EDST	Treatment	2,27	4.570	**0.019**
			Time	6,162	24.146	**0.000**
			Time × treatment	12,162	1.099	0.363
		N# of EDFS	Treatment	2,27	1.070	0.356
			Time	6,162	7.706	**0.000**
			Time × treatment	12,162	1.255	0.250
		N# of ESFL	Treatment	2,27	2.325	0.116
			Time	6,162	3.054	**0.007**
			Time × treatment	12,162	0.879	0.568
		Average latency to escape	Treatment	2,36	2.545	*0.092*
			Time	4,144	3.009	**0.020**
			Time × treatment	8,144	0.406	0.916
	Reversal learning	N# of EDST	Treatment	2,36	0.330	0.720
			Time	2,72	10.168	**0.000**
			Time × treatment	4,72	0.713	0.585
		N# of ESFL	Treatment	2,36	0.320	0.727
			Time	2,72	10.593	**0.000**
			Time × treatment	4,72	0.741	0.566
	Learning + reversal learning	N# of EDST	Treatment	2,27	7.555	**0.002**
			Time	9,243	16.859	**0.000**
			Time × treatment	18,243	0.818	0.678
		N# of ESFL	Treatment	2,27	3.124	*0.060*
			Time	9,243	49.196	**0.000**
			Time × treatment	18,243	1.066	0.388

Changes between last learning day and first reversed learning day analyzed by one way ANOVA						
Experiment	Parameters	Effect	Degree of freedom	F	p	
Operant conditioning	Total responses	Treatment	2,35	0.882	0.422	
	Reward preference	Treatment	2,35	0.364	0.697	
Active avoidance	N# of EDST	Treatment	2,23	0.540	0.589	
	N# of ESFL	Treatment	2,13	0.575	0.576	

EDST: escape during stimulus; EDFS: escape during footshock; ESFL: escape failure; N.A. not applicable.

The subgroups of mice bearing no DREADD sequence (Control), GABA stimulatory sequence (Stim group) and GABA inhibitory sequence (Inhib group) needed different time to learn the task (preference of baited nose hole exceeded the chance level of 50% by single sample t-test) (Fig. 4c, Suppl. Table 2). The mice of the Control group learned the paradigm by the 4th day of the experiment, those of the Stim group by the 10th day, and the mice of the Inhib group by the 5th day. There were no significant differences between the groups during the learning phase and the group × time interaction did not reach the level of significance either (Table 2).

On days 11–17, during the reversal learning phase, there were significant differences in total responses between the Stim group and the other groups, as the responses dropped for the Control and Inhib groups, while they remained unchanged for the Stim group (p = 0.027; Fig. 4b, Table 2). Moreover, during this phase the mice in the Stim group showed higher preference for the rewarded nose hole, and this was the only group that consistently exceed the chance level of 50% after the 12th day. When we expressed the changes between the last day of learning and first day of reversal learning, there were no significant difference in reward preference and total responses between the groups (Fig. 4d, Table 2).

For assessment of impulsivity, we analyzed timeout responses as well, which showed treatment effect during the reversal learning phase with significantly higher levels in stimulatory as both the control and inhibitory groups (p < 0.05) (Fig. 4e, Table 2). The number of all rewarded or non-rewarded responses (either baited or timeout) showed only time effect both during the learning and reversal learning phases (data not shown).

#### Active avoidance

**Figure 5:**
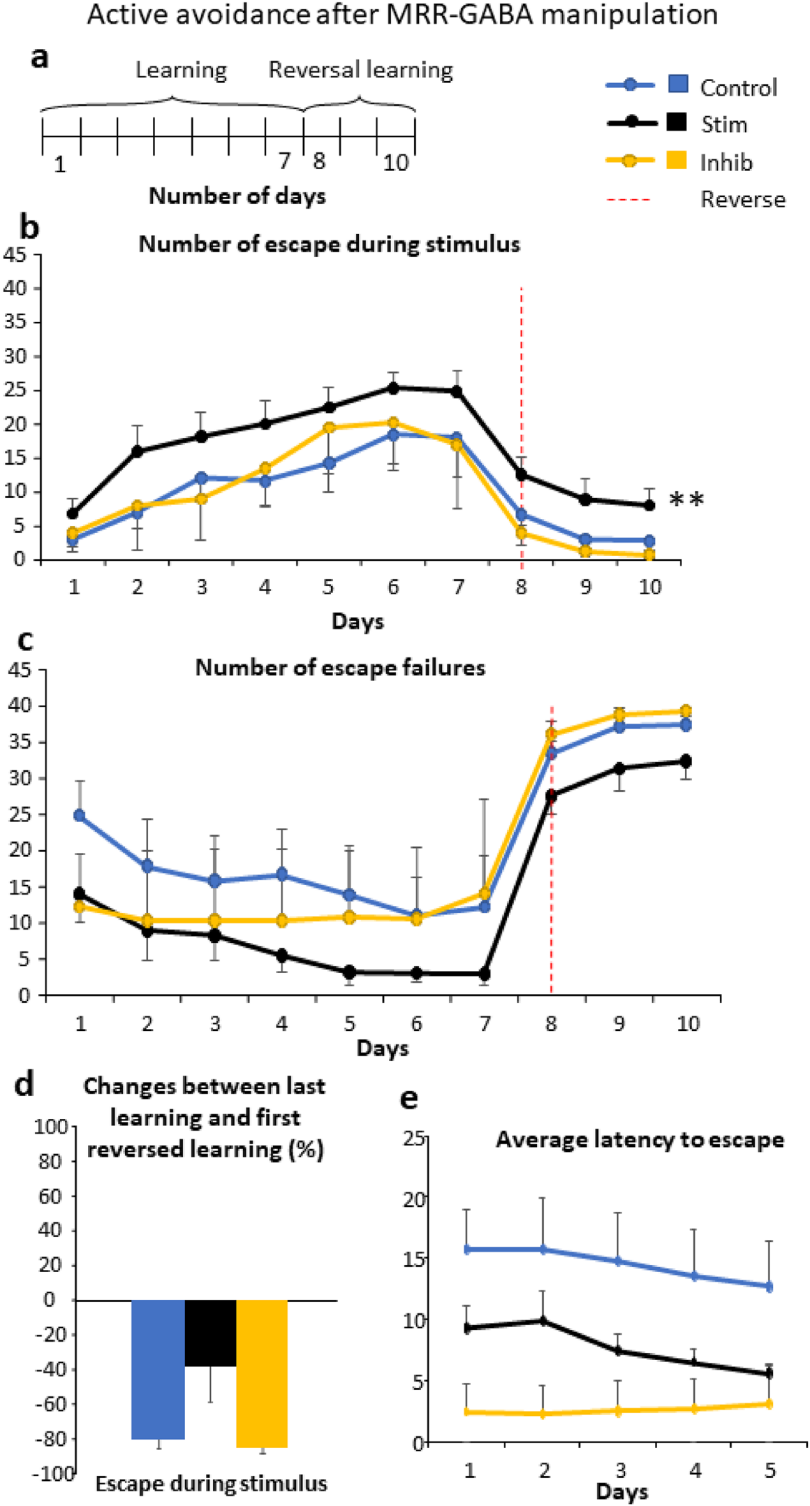
Active avoidance after manipulation of the GABAergic cells of the median raphe region (Experiment 2). (**a**) Schematic timeline of the active avoidance test, carried out similarly as during Experiment 1. The learning phase lasted for 7 days, while the reversal learning phase was 3-day-long. (**b**) The number of escapes during stimulus increased throughout the learning phase and the stimulatory group had higher total responses. During reversal learning phase (indicated by red dashed lines), there were no differences between the groups. (**c**) There were no significant differences in the number of escape failures between the groups. (**d**) Percentage of change between last day of learning phase and first day of reversal learning did not differ across the groups in the case of escape stimulus. (**e**) Average escape latency during learning as a possible measure of impulsivity. Data are expressed as mean ± SEM. Data were compared to each other with repeated-measures ANOVA (escapes during stimulus, escapes during footshock, escape failures) and t-test (change between last and first). ^#^p < 0.01 main treatment effect during Bonferroni posthoc comparison, stimulatory group vs. control as well as inhibitory groups. Inhib: inhibitory receptor sequence containing virus vector, MRR: median raphe region, Stim: stimulatory receptor sequence containing virus vector.

During the 7 days of learning the number of EDST increased in all groups (Fig. 5b, Table 2). The Stim group showed higher total responses than the other two groups (p < 0.05). Complementary to this, EDFS (data not shown) and the number of the escape failures (Fig. 5c) decreased gradually during the learning.

During the reversal learning phase (days 8–10) the number of EDST decreased across the days (Fig. 5b, Table 2). Similar to the results observed in experiment 1, the number of escape failures increased in all groups throughout the reversal training days. Take into consideration the whole observation period the ADST difference was even more pronounced being highly different in Stim than in Control and Inhib groups (p < 0.01) (Fig. 5b, Table 2). No significant differences were found between the groups in the changes when the last day of learning was compared with the first day of reversal learning (Fig. 5d, Table 2).

As a possible sign of impulsivity, the average latency to escape was also analyzed without any significant difference between the treatment groups (Fig. 5e, Table 2).

## Discussion

Despite extensive research on the MRR^11,12,17^ a consensual understanding of its involvement in learning has remained elusive. The present study shows that manipulating the whole MRR had no influence on operant and active avoidance learning nor reversal learning, while the stimulation of the MRR GABAergic neurons increased learning in the active avoidance paradigm and enhanced total responses in the operant conditioning task (Table 3).

**Table 3:** Summary of the results.

Experiment 1. Whole median raphe stimulation				
Experiment	Phase	Parameters	Excitatory	
Operant conditioning	Learning	Reward preference	Ø	
		Total response	Ø	
	Reversal learning	Reward preference	Ø	
		Total response	Marginally decreased	
Active avoidance	Learning	N# of EDST	Ø	
		N# of EDFS	Ø	
		N# of ESFL	Ø	
	Reversal learning	N# of EDST	Ø	
		N# of EDFS	Ø	
		N# of ESFL	Marginally increased	

Experiment 2. Manipulation of the VGAT positive cells of the median raphe region				
Experiment	Phase	Parameters	Excitatory	Inhibitory
Operant conditioning	Learning	Reward preference	Ø	Ø
		Total response	Ø	Ø
	Reversal learning	Reward preference	Increased	Ø
		Total response	Increased	Ø
Active avoidance	Learning	N# of EDST	Increased	Ø
		N# of EDFS	Ø	Ø
		N# of ESFL	Ø	Ø
	Reversal learning	N# of EDST	Ø	Ø
		N# of EDFS	Ø	Ø
		N# of ESFL	Ø	Ø

Ø no difference compared to control; EDST: escape during stimulus; EDFS: escape during footshock; ESFL: escape failure.

In the present experiment using an adenoassociated virus vector (AAV) containing only synapsin promoter without the Cre-loxP system, theoretically all neurons should have been labelled by RFP in proportion of their prevalence. Indeed, the vast majority of observed infected cells, 43.1% were GABAergic. This is in accordance with the previously reported predominance of GABAergic neurons (65.7 ± 4.38%)^16^. However, much less serotoninergic (only 1.89% TPH positive in contrast to previously reported 9.6%) and VGluT3 positive (8.31% in contrast previously reported 11.4%; partly overlapping with TPH) cells were co-labelled with RFP^16^. As AAVs were expressed differently in different cell-types not completely overlapping with their previously reported prevalence, we might conclude that AAVs might have some tropism and even when we intended to use non-cell-type specific manipulation, we might selectively influence special cell populations.

As in Experiment 1. the share of serotonergic and glutamatergic neurons was very small; stimulation affected the MRR primarily via the GABAergic system. However, the major differences in the behavioral consequences of non-specific (Experiment 1) and GABA-specific (Experiment 2) stimulation suggest that either the few glutamatergic and serotonergic neurons were able to counteract the effects of the large number of GABAergic neurons, or that the latter was achieved by the stimulation of unidentified neurons. Indeed, a subsequent study by an overlapping set of authors suggested the presence of a large, VGLUT2 positive glutamatergic neuron population in the MRR^18^. Based on these earlier studies, the total share of glutamatergic neurons expressing either VGLUT3 or VGLUT2 is close to the share of GABAergic neurons in the MRR. As such, we hypothesize that the effects of GABAergic stimulation were counteracted in Experiment 1 by the concurrent stimulation of glutamatergic neurons. By contrast, the effects of GABAergic stimulation became conspicuous in Experiment 2, where the stimulation was specific.

Indeed, the specific stimulation of MRR-GABA cells induced significant changes in both learning paradigms used. In the operant conditioning test, mice bearing GABA stimulatory sequence showed a high response rate even after the start of the reversal learning phase. This suggests a proclivity to impulsive behavior^19^. In support the number of timeout responses was also increased after stimulation, however, long time treatment was necessary (from 11th days on) and the unaffected escape latency during active avoidance test did not suggest a general increase in impulsivity, either. Additional, more specific studies is required to assess impulsivity. Our major question was the effect on memory formation, which we found to be negligible. Operant conditioning is based on reward, and the reward response is commonly associated with the mesolimbic dopaminergic system^20^. Over the past years, it was demonstrated that the ventral tegmental area (VTA)—one of the components of the mesolimbic dopaminergic system—does not contain only dopaminergic neurons, but also GABAergic cells^21^. Additionally, in recent retrograde tracing studies it has been documented that GABAergic neurons originating in the MRR have modest projections to the VTA^22^, silencing not only local interneurons but also other brain regions^23^. By doing so, they may act—among others—as a gate of dopaminergic activity, mediating the response to reward and aversion, and—in our case—impulsivity, in which dopaminergic VTA neurons are also implicated^24^.

In the active avoidance test, mice bearing GABA stimulatory sequence displayed a high escape rate during the stimulus, resulting in a maladaptive and excessive avoidance coping response. These results indicate an increased formation of aversive memory during the stimulation of the GABAergic cells. This corroborates with the suggestion that MRR actively participates in the regulation of negative memories^18^. Such behavior was not observed during the reversal learning phase, in which all mice performed similarly. However, the footshock is a rather excessive motivation, and the response during the reversal learning phase is passive (the animals do not have to leave the chamber). It is therefore might have been difficult to detect any differences.

We confirmed previous results that chemogenetics is an effective method to manipulate the neuron populations of the MRR, as we observed CNO-induced elevation in the c-Fos expression^25,26^. It was important as previous studies questioned whether CNO reached the brain in functionally relevant concentration^26^. As original description considered CNO as inert drug^27,28^**,** we used saline as control for Experiment 1. Although subsequent studies suggested possible back-metabolism of CNO to clozapine^26^, methodological issues could hardly influence the ineffectiveness of whole MRR manipulation. Moreover, we would have expected that the excitatory and inhibitory groups in Experiment 2. would have opposite effects, but our findings did not support this idea. The explanation could be that the two kinds of DREADD sequences activate different cellular pathways (Gq and Gi). Also, stimulation is a more active process, while inhibition mainly reduces the impact of other stimulatory signals. A further limitation of our technique was that it is hard to target MRR without going through the DR, thereby in many cases cells of both regions were infected. Although there were no statistically significant difference between the behavior of exclusively MRR targeted and MRR + DR co-targeted animals (Supplementary Table 1), we should be aware of the important role of serotonin and especially DR in reversal learning^29,30^.

## Conclusion

We have demonstrated that the stimulation of the MRR-GABA neurons in VGAT-Cre mice reduced reversal learning without an effect on memory formation during the operant conditioning test. Moreover, the same chemogenetic manipulation increased the formation of negative memory during the active avoidance test. The ineffectiveness of the whole MRR stimulation might be partially due to the limitation of the chemogenetic techniques, but underlines the importance of cell-type specific manipulation. Further studies addressing GABAergic subpopulations in the MRR may provide additional insights into the formation of reward- and punishment related memories.

## Methods

### Animals

All mice (C57BL/6J background) were obtained from the local colonies of the Institute of Experimental Medicine, Budapest, Hungary. VGAT-Cre mice (origin: The Jackson Laboratory, #016962) were bred in homozygous mating pairs. During the test battery performance adult male mice (14–15-week-old) were housed in groups of 2–3 in Macrolon cages (40 cm × 25 cm × 26 cm) under a 12-h light–dark cycle (lights on at 7 p.m., 21 ± 1 °C, 50–60% humidity), with food (standard mice chow, Charles River, Hungary) and tap-water available ad libitum if not stated otherwise. The tests were conducted during the early dark, active phase.

All experiments were approved by the Workplace Animal Welfare Committee of Institute of Experimental Medicine and National Scientific Ethical Committee on Animal Experimentation of Hungary (PEI/001/33-4/2013, PE/EA/254-7/2019) and performed according to the European Communities Council Directive recommendations for the care and use of laboratory animals (2010/63/EU). The authors complied with the ARRIVE guidelines.

### Experimental design

The C57BL/6J and VGAT-Cre animals were tested in separate series with minor differences in the protocol.

#### Experiment 1: Whole MRR manipulation

C57BL/6J control (N = 6) and MRR-stimulated (N = 9) animals were used. All animals were injected with an AAV containing stimulatory DREADD^31^ and RFP sequences into their MRR^26^. The animals had 4 weeks to recover from the surgery, during which they were accustomed to the reversed light–dark cycle (min 2 weeks). Then an operant conditioning experiment with 4 days habituation to reduced food accessibility (to maintain their body weight on 80% of their initial weight), 14 days learning and 7 days reversed learning phases (Fig. 2a) was conducted followed by 4 days recovery and 5 days learning and 5 days reversed learning in an active avoidance paradigm (Fig. 3a) as we described earlier^32^. On each test day, 30 min before the animals were put into the testing box an intraperitoneal (i.p.) injection of either saline (control) or CNO (1 mg/10 ml saline/kg) was delivered. At the termination of the experiment the anesthetized animals were transcardially perfused 2 h after the CNO injection and their brains were checked by RFP immunohistochemistry for correctness of the injection as well as for detailed determination of the infected cell-types. We already successfully confirmed that the cells of the MRR express the RFP suggesting that they also express the DREADD receptor^26^. Only mice with correct hits were included in further analysis. The results of mice having out of target labelling in their dorsal raphe were not different from exclusively MRR-targeted animals, therefore their data were merged (Suppl. Tables 1 and 2, Suppl. Figs. 1 and 2).

#### Experiment 2: MRR-GABA manipulation

As Experiment 1 showed that most of the infected cells were GABAergic, we conducted a further experiment using VGAT-Cre mice. We followed the steps of Experiment 1 (Figs. 4a and 5a) using different viruses containing control (n = 11), stimulatory (n = 15) and inhibitory (n = 13) sequences^18^. In this experiment all animals got CNO injections. It was confirmed previously that this technique sufficiently manipulates MRR-GABA cells^25^.

### Delivering AAVs into the MRR

Mice were anaesthetized (0.1 ml/10 g mixture of 0.5 ml ketamine [Produlab Pharma B.V.], 0.1 ml xylazine [Produlab Pharma B.V.] and 2.4 ml saline [KabiPac]) and with the help of a stereotaxic frame (David Kopf Instruments, Tujunga, CA, USA) and nanoinjector AAVs (10 nl; Addgene) were injected into the MRR (AP: − 4.1 mm; L: 0 mm; DV: 4.6 mm from Bregma) with the help of a glass micropipette as described earlier,^18,26^. During Experiment 1, all animals got the same virus (AAV2-hSyn-hM3Dq-mCherry, 3.0e12 GC/ml titer, #50474). For Experiment 2, three subgroups were formed based on the injected Cre-dependent AAVs containing different DREADD sequences: control (no DREADD sequence, only RFP, AAV8-hSyn::DIO-mCherry, 4.1e12 GC/ml titer, #50459), stimulatory (AAV8-hSyn::DIO-hM3Dq-mCherry, 4.0e12 GC/ml titer, #44361) and inhibitory (AAV8-hSyn::DIO-hM4Di-mCherry, 1.9e13 GC/ml titer, #44362).

### Behavioral testing

Tests were carried out between 9 and 13 h (early dark phase) in a separate room under similar lighting condition as in the animal facility and measured automatically by the equipments for operant chamber or active avoidance (Med Associates, St. Albans, VT, USA). The chambers were placed inside sound-attenuating cubicles and were interfaced with a computer running Med-PC IV software. Six animals were tested in one run containing animals from each group. Each test apparatus was cleaned with 20% ethanol and water and dried prior the next animal was introduced. The test battery included two types of reinforcement-based learning paradigms. In both tests reversal learning was also assessed, which was evaluated during the reversal learning phase.

#### Operant conditioning

To increase motivation the mice were kept on restricted diet started 72 h before testing^32^. The test was performed in an automated operant chamber using 45 mg food pellets (Bio-Serv Dustless Precision Rodent Pellet, Bilaney Consultants GmbH, Germany) as reward^33^. Animals were placed inside a test chamber for 30 min and were allowed to freely explore the environment. One of the nose pokes was immediately associated with a reward followed by a 25 s long timeout with the chamber light switched on. During the timeout period, responses were not rewarded, but were registered and used as a marker of impulsivity^34^.

There were small differences between the experiment 1 and experiment 2. In the experiment 1, the test was divided into two phases, namely learning (day 1–14) and reversal learning (reversed learning, day 15–21) (Fig. 2a). In the experiment 2, the learning phase lasted 10 days and that of reversal learning 7 days (Fig. 4a). The position of the baited nose poke was changed between the phases in both experiments.

Reward preference (ratio of responses on the rewarded nose poke) was calculated as follows:

$$Reward \; preference= \frac{correct \; nose \; poke}{incorrect+correct \; nose\; pokes}\times 100$$

and the total number of responses (correct + incorrect) was also recorded.

#### Active avoidance (shuttle-box) test

Classical automated shuttle-box apparatus consisted of two identical compartments with photobeam sensors, stimulus light, tone generator, stainless steel grid floor and a guillotiner door^35^.

Mice were placed in the left or right compartment of the apparatus for 10 days. After 1 min of habituation the 40 trials (each 30 s long) started. In each trial, 20 s after the start the light turned on and a tone was played, meanwhile the guillotine door opened (conditioning stimuli). During the last 5 s of each trial an electric footshock (0.15 mA) was applied to the grid floor (unconditioned stimulus) of one of the compartments. At the end of the trial all stimuli were switched off, the guillotine door closed and 5 s intertrial interval (ITI) started, then the subsequent trial was conducted. The 5 (Experiment 1, Fig. 3a) or 7 (Experiment 2, Fig. 5a) days learning phase was immediately followed by 5 days (day 6–10 in Experiment 1) or 3 days (day 8–10 in Experiment 2) of reversal learning phase, in which the shocks were applied to the opposite compartment.

An avoidance response was recorded when the animal avoided the electric shock by entering (or during the reversed phase: not entering) the other compartment during the conditioned stimuli (escape during stimulus—EDST) or during the footshock (escape during footshock—EDFS). Escape failure (ESFL) was recorded when the animals remained in the chamber and got footshock (or during reversal phase: jumped into the other compartment). Average escape latencies were also calculated as possible sign of impulsivity. Due to missing data we present only the first 5 days of learning for both experiment.

### Immunohistochemistry and microscopy

To check the correctness of the AAV injection, a nickel-3,3′-diaminobenzidine (Ni-DAB) staining against RFP was conducted^36^. The slices were washed with phosphate buffered saline (PBS) for 3 × 10 min. Membranes were permeabilized by adding 0.5% Triton X-100 (TXT) and 0.3% H_2_O_2_, followed by 2 × 10 min PBS washing. Blocking was done by 2% bovine serum albumin (BSA) diluted in PBS for 1 h. The slices were incubated in anti-RFP primer solution (1:4000, rabbit; 600-401-379, 2% BSA; 0.1% TXT diluted in PBS) for 2 nights on 4 °C. After 3 × 10 min of PBS washing they were incubated in biotinylated (biotin-SP) anti-rabbit secondary antibody solution (1:100. donkey; 2% BSA diluted in PBS). After 10 min PBS, then 10 min TRIS washing the slices were kept in avidin–biotin complex (ABC) diluted in TRIS for 1 h. They were pre-incubated in the dilution of TRIS, DAB (10 mg/ml) and 1% NiNH_4_SO_4_ for 10 min. After adding 0.003% H_2_O_2_ and waiting for precipitation the slices were washed with TRIS for 10 min. They were mounted with gelatin, dehydrated in xylol and covered with DPX (Sigma-Aldrich).

The Ni-DAB-stained slices were evaluated with Olympus DP70 light microscope (4× objective). The virus expression was examined from − 4.04 to − 4.96 mm from Bregma. If there was no staining, or it was unilateral, or other brain regions were also stained, then the test animal and the data belonging to it was excluded from the statistical analysis. In cases where both the MRR and dorsal raphe were hit, statistical analysis was done to verify if the hits on dorsal raphe affected the behavior.

To verify which cell-types were infected in Experiment 1, double immunofluorescent staining was done. The slices were washed with PBS for 3 × 10 min. Blocking was done with 5% normal goat serum (NGS, #31873, Thermo Fisher Scientific, Waltham, MA, US) and 0.2% TXT diluted in PBS for 30 min. For 2 nights they were incubated in anti-RFP (1:1000, rabbit), anti-GABA (1:500, rabbit, A2052, GABAergic marker) or anti-tryptophan hydroxylase (1:500, mouse, T0678, TPH, enzyme in serotoninergic cells) or anti-vesicular glutamate transporter 3 (1:500, rabbit, 135203, VGluT3, a major glutamatergic marker^16^), 5% NGS and 0.2% TXT primer solution diluted in PBS on 4 °C. After 3 × 10 min of PBS washing they were incubated in a seconder solution of anti-rabbit conjugated with Alexa-488 (1:500, goat) and anti-rat conjugated with Alexa-594 (1:1000, goat) diluted in PBS. After 3 × 10 min of PBS washing the slices were mounted with gelatin and covered with Mowiol. The double immunofluorescent staining was evaluated with C2 confocal laser-scanning microscope (Nikon Europe; 20× objective).

c-Fos immunohistochemistry was applied to assess possible chemogenetic manipulation-induced neuronal activity in MRR. After 3 × 10 min PBS washing and 30 min incubation in 10% NGS fluorescent immunolabeling was used against c-Fos and RFP (1:2000 guinea-pig polyclonal anti-c-Fos IgG, #226004, Synaptic Systems with monoclonal rabbit anti-RFP IgG 1:4000, #600-401-379, Rockland) diluted in 2% NGS with 0.1% TXT in PBS overnight at 4 °C. Primary antibodies were detected by fluorescent-conjugated antibodies (1:500 Alexa-488 conjugated donkey anti-guinea-pig, #S32354, ThermoFisher Scientific, Waltham, MA, USA, and 1:500 A-594 conjugated goat anti-rabbit, #ab150160, Abcam plc, Cambridge, UK). c-Fos-RFP immunohistochemistry was imaged by C2 Confocal Laser-Scanning Microscope (Nikon CFI Plan Apo VC20X/N.A. 0.75, xy:0.62 μm/pixel, Nikon Europe, Amsterdam, The Netherlands). Quantitative analysis of the colocalizations was done with the NIS Elements software (Nikon Europe, Amsterdam, Netherlands).

### Statistical analysis

Data were analyzed by Statistica 13.0 (StatSoft Inc., Tulsa, USA) utilizing single sample t-test (operant conditioning in comparison to 50%), one-way analysis of variance (ANOVA) (operant conditioning), repeated measure ANOVA (operant conditioning, active avoidance) followed by Bonferroni posthoc comparison where appropriate. Data are expressed as mean ± SEM and p < 0.05 was considered statistically significant.

### Supplementary Information

Supplementary Table 1.

Supplementary Table 2.

Supplementary Table 3.

Supplementary Table 4.

Supplementary Figure 1.

Supplementary Figure 2.

## Data Availability

The datasets generated during and/or analyzed during the current study are available from the corresponding author on reasonable request.
