# Supplementary material for: The role of the GABAergic cells of the median raphe region in reinforcement-based learning
Source: Sci Rep. 2024 Jan 12;14:1175. doi: 10.1038/s41598-024-51743-y (PMC10786920; doi:10.1038/s41598-024-51743-y)
Supplement: Supplementary file 1 — Supplementary Table 1. [file 41598_2024_51743_MOESM1_ESM.docx]

**Supplementary Table 1.** Statistical details for the whole median stimulation (Experiment 1.)

Reward preference in operant conditioning– single sample t test

1. Control group to 50%

| Day | t-value | Df | p |
| --- | --- | --- | --- |
| 1 | -2.253 | 5 | 0.074 |
| 2 | -0.019 | 5 | 0.986 |
| 3 | 0.763 | 5 | 0.480 |
| 4 | -0.091 | 5 | 0.931 |
| 5 | 1.385 | 5 | 0.225 |
| 6 | 4.346 | 5 | 0.007 |
| 7 | 1.934 | 5 | 0.111 |
| 8 | 0.325 | 5 | 0.759 |
| 9 | 1.542 | 5 | 0.184 |
| 10 | 2.025 | 5 | 0.099 |
| 11 | 2.982 | 5 | 0.031 |
| 12 | 3.743 | 5 | 0.013 |
| 13 | 3.032 | 5 | 0.029 |
| 14 | 2.531 | 5 | 0.052 |
| 15 | -6.502 | 5 | 0.001 |
| 16 | -2.056 | 5 | 0.095 |
| 17 | 0.159 | 5 | 0.880 |
| 18 | 0.153 | 5 | 0.884 |
| 19 | 1.557 | 5 | 0.180 |
| 20 | 0.846 | 5 | 0.436 |
| 21 | 0.449 | 5 | 0.672 |

B CNO group to 50%

| Day | t-value | df | p |
| --- | --- | --- | --- |
| 1 | -1.282 | 8 | 0.236 |
| 2 | -0.084 | 8 | 0.935 |
| 3 | 2.424 | 8 | 0.042 |
| 4 | 3.286 | 8 | 0.011 |
| 5 | 1.730 | 8 | 0.122 |
| 6 | 1.039 | 8 | 0.329 |
| 7 | 1.897 | 8 | 0.094 |
| 8 | 1.281 | 8 | 0.236 |
| 9 | 2.193 | 8 | 0.060 |
| 10 | 0.683 | 8 | 0.514 |
| 11 | 1.864 | 8 | 0.099 |
| 12 | 1.999 | 8 | 0.081 |
| 13 | 1.392 | 8 | 0.201 |
| 14 | 1.521 | 8 | 0.167 |
| 15 | -6.137 | 8 | 0.000 |
| 16 | -0.392 | 8 | 0.706 |
| 17 | 0.812 | 8 | 0.441 |
| 18 | 0.620 | 8 | 0.552 |
| 19 | 2.176 | 8 | 0.061 |
| 20 | 2.650 | 8 | 0.029 |
| 21 | 0.172 | 7 | 0.869 |
