# Supplementary material for: The role of the GABAergic cells of the median raphe region in reinforcement-based learning
Source: Sci Rep. 2024 Jan 12;14:1175. doi: 10.1038/s41598-024-51743-y (PMC10786920; doi:10.1038/s41598-024-51743-y)
Supplement: Supplementary file 2 — Supplementary Table 2. [file 41598_2024_51743_MOESM2_ESM.docx]

**Supplementary Table 2.** Statistical details for the GABAergic cell manipulation of the median raphe region (MRR) (Experiment 2.) Reward preference in operant conditioning– single sample t test.

1. Control group to 50%

| Day | t-value | df | p |
| --- | --- | --- | --- |
| 1 | 2.362 | 10 | 0.040 |
| 2 | 0.554 | 10 | 0.592 |
| 3 | 0.996 | 10 | 0.343 |
| 4 | 2.367 | 10 | 0.039 |
| 5 | 3.721 | 10 | 0.004 |
| 6 | 4.759 | 10 | 0.001 |
| 7 | 4.966 | 10 | 0.001 |
| 8 | 4.712 | 10 | 0.001 |
| 9 | 5.841 | 10 | 0.000 |
| 10 | 5.019 | 10 | 0.001 |
| 11 | -0.282 | 10 | 0.784 |
| 12 | 1.520 | 10 | 0.159 |
| 13 | 1.268 | 10 | 0.234 |
| 14 | 1.550 | 10 | 0.152 |
| 15 | 2.193 | 9 | 0.056 |
| 16 | 2.075 | 9 | 0.068 |
| 17 | 0.459 | 9 | 0.657 |

1. Stimulatory group to 50%

| Day | t-value | df | p |
| --- | --- | --- | --- |
| 1 | -0.238 | 13 | 0.816 |
| 2 | -0.896 | 13 | 0.387 |
| 3 | -0.565 | 13 | 0.582 |
| 4 | 0.307 | 13 | 0.764 |
| 5 | 1.176 | 13 | 0.261 |
| 6 | 1.077 | 13 | 0.301 |
| 7 | 2.144 | 13 | 0.052 |
| 8 | 1.925 | 13 | 0.076 |
| 9 | 3.013 | 13 | 0.010 |
| 10 | 5.988 | 13 | 0.000 |
| 11 | 0.349 | 13 | 0.733 |
| 12 | 2.913 | 13 | 0.012 |
| 13 | 3.806 | 13 | 0.002 |
| 14 | 4.724 | 12 | 0.000 |
| 15 | 2.759 | 13 | 0.016 |
| 16 | 3.118 | 12 | 0.009 |
| 17 | 3.185 | 12 | 0.008 |

1. Inhibitory group to 50%

| Day | t-value | df | p |
| --- | --- | --- | --- |
| 1 | -0.306 | 12 | 0.765 |
| 2 | -1.184 | 12 | 0.259 |
| 3 | -1.190 | 12 | 0.257 |
| 4 | -0.236 | 12 | 0.817 |
| 5 | 2.340 | 12 | 0.037 |
| 6 | 2.511 | 12 | 0.027 |
| 7 | 4.414 | 12 | 0.001 |
| 8 | 5.167 | 12 | 0.000 |
| 9 | 4.003 | 12 | 0.002 |
| 10 | 4.296 | 12 | 0.001 |
| 11 | -2.667 | 12 | 0.021 |
| 12 | 1.470 | 12 | 0.167 |
| 13 | 3.032 | 12 | 0.010 |
| 14 | 1.820 | 12 | 0.094 |
| 15 | 1.029 | 12 | 0.324 |
| 16 | 3.361 | 12 | 0.006 |
| 17 | 1.186 | 12 | 0.258 |
