# Supplementary material for: The role of the GABAergic cells of the median raphe region in reinforcement-based learning
Source: Sci Rep. 2024 Jan 12;14:1175. doi: 10.1038/s41598-024-51743-y (PMC10786920; doi:10.1038/s41598-024-51743-y)
Supplement: Supplementary file 3 — Supplementary Table 3. [file 41598_2024_51743_MOESM3_ESM.docx]

**Supplementary Table 3.** Statistical details for the whole median stimulation (Experiment 1.)

1. Operant conditioning analyzed by Repeated Measures ANOVA, comparison of MRR and MRR+DR infected groups

| Phase | Parameters | Effect | df | F | p |
| --- | --- | --- | --- | --- | --- |
| Learning | Reward preference | Treatment | 1,7 | 0.749 | 0.415 |
|  |  | Time | 13,91 | 1.146 | 0.331 |
|  |  | Time x Treatment | 13.91 | 0.490 | 0.924 |
|  | Total responses | Treatment | 1,7 | 4.311 | 0.075 |
|  |  | Time | 13,91 | 3.287 | 0.000 |
|  |  | Time x Treatment | 13,91 | 1.123 | 0.350 |
| Reversal learning | Reward Preference | Treatment | 1,6 | 1.822 | 0.225 |
|  |  | Time | 6,36 | 4.522 | 0.001 |
|  |  | Time x Treatment | 6,36 | 0.170 | 0.983 |
|  | Total responses | Treatment | 1,6 | 0.218 | 0.656 |
|  |  | Time | 6,36 | 0.773 | 0.595 |
|  |  | Time x Treatment | 6,36 | 0.183 | 0.975 |

1. Active avoidance analyzed by Repeated Measures ANOVA, comparison of MRR and MRR+DR infected groups

| Phase | Parameters | Effect | df | F | p |
| --- | --- | --- | --- | --- | --- |
| Learning | N# of EDST | Treatment | 1,7 | 0.617 | 0.810 |
|  |  | Time | 4,28 | 12.011 | 0.000 |
|  |  | Time x Treatment | 4,28 | 0.603 | 0.662 |
|  | N# of EDFS | Treatment | 1,7 | 0.781 | 0.405 |
|  |  | Time | 4,28 | 3.761 | 0.014 |
|  |  | Time x Treatment | 4,28 | 2.720 | 0.049 |
|  | N# of ESFL | Treatment | 1,7 | 0.739 | 0.418 |
|  |  | Time | 4,28 | 0.845 | 0.508 |
|  |  | Time x Treatment | 4,28 | 2.266 | 0.087 |
| Reversal learning | N# of EDST | Treatment | 1,7 | 0.072 | 0.795 |
|  |  | Time | 4,28 | 17.754 | 0.000 |
|  |  | Time x Treatment | 4,28 | 1.040 | 0.404 |
|  | N# of EDFS | Treatment | 1,7 | 0.046 | 0.836 |
|  |  | Time | 4,28 | 0.077 | 0.988 |
|  |  | Time x Treatment | 4,28 | 1.046 | 0.401 |
|  | N# of ESFL | Treatment | 1,7 | 0.075 | 0.791 |
|  |  | Time | 4,28 | 5.884 | 0.001 |
|  |  | Time x Treatment | 4,28 | 0.576 | 0.682 |

Abbreviations: CNO: clozapine-N-oxid; DR: dorsal raphe; EDST: Escape during stimulus; EDFS: Escape during footshock; ESFL: Escape failure; MR: median raphe
