# Supplementary material for: The role of the GABAergic cells of the median raphe region in reinforcement-based learning
Source: Sci Rep. 2024 Jan 12;14:1175. doi: 10.1038/s41598-024-51743-y (PMC10786920; doi:10.1038/s41598-024-51743-y)
Supplement: Supplementary file 4 — Supplementary Table 4. [file 41598_2024_51743_MOESM4_ESM.docx]

**Supplementary Table 4.** Statistical details for manipulating the GABAergic cells of the median raphe (Experiment 2). Comparison of MRR and MRR+DR stimulation by Repeated Measures ANOVA.

1. Operant conditioning

| \Phase | Parameters | Groups | Effect | df | F | p |
| --- | --- | --- | --- | --- | --- | --- |
| Learning | Reward Preference | Stim MRR / Stim MRR + DR | Treatment | 1,12 | 1.064 | 0.322 |
|  |  |  | Time | 9,108 | 5.047 | 0.000 |
|  |  |  | Time x Treatment | 9,108 | 0.626 | 0.772 |
|  |  | Inhib MRR / Inhib MRR + DR | Treatment | 1,12 | 0.147 | 0.708 |
|  |  |  | Time | 9,108 | 8.69 | 0.000 |
|  |  |  | Time x Treatment | 9,108 | 0.400 | 0.932 |
|  | Total responses | Stim MRR / Stim MRR + DR | Treatment | 1,12 | 0.239 | 0.633 |
|  |  |  | Time | 9,108 | 5.915 | 0.000 |
|  |  |  | Time x Treatment | 9,108 | 0.599 | 0.795 |
|  |  | Inhib MRR / Inhib MRR + DR | Treatment | 1,12 | 2.943 | 0.114 |
|  |  |  | Time | 9,108 | 8.495 | 0.000 |
|  |  |  | Time x Treatment | 9,108 | 0.917 | 0.513 |
|  | Timeout Rewards | Stim MRR / Stim MRR + DR | Treatment | 1,12 | 0.239 | 0.633 |
|  |  |  | Time | 9,108 | 5.915 | 0.000 |
|  |  |  | Time x Treatment | 9,108 | 0.599 | 0.795 |
|  |  | Inhib MRR / Inhib MRR + DR | Treatment | 1,12 | 2.943 | 0.114 |
|  |  |  | Time | 9,108 | 8.495 | 0.000 |
|  |  |  | Time x Treatment | 9,108 | 0.917 | 0.513 |
|  | Timeout Non-Rewards | Stim MRR / Stim MRR + DR | Treatment | 1,12 | 0.007 | 0.931 |
|  |  |  | Time | 9,108 | 0.727 | 0.682 |
|  |  |  | Time x Treatment | 9,108 | 1.612 | 0.120 |
|  |  | Inhib MRR / Inhib MRR + DR | Treatment | 1,12 | 0.094 | 0.764 |
|  |  |  | Time | 9,108 | 1.222 | 0.289 |
|  |  |  | Time x Treatment | 9,108 | 0.223 | 0.990 |
| Reversal learning | Reward Preference | Stim MRR / Stim MRR + DR | Treatment | 1,11 | 4.116 | 0.067 |
|  |  |  | Time | 6,66 | 3.870 | 0.002 |
|  |  |  | Time x Treatment | 6,66 | 0.051 | 0.999 |
|  |  | Inhib MRR / Inhib MRR + DR | Treatment | 1,11 | 0.075 | 0.788 |
|  |  |  | Time | 6,66 | 4.933 | 0.000 |
|  |  |  | Time x Treatment | 6,66 | 0.227 | 0.966 |
|  | Total responses | Stim MRR / Stim MR + DR | Treatment | 1,11 | 0.102 | 0.754 |
|  |  |  | Time | 6,66 | 0.621 | 0.712 |
|  |  |  | Time x Treatment | 6,66 | 0.171 | 0.983 |
|  |  | Inhib MRR / Inhib MRR + DR | Treatment | 1,11 | 1.097 | 0.317 |
|  |  |  | Time | 6,66 | 1.788 | 0.114 |
|  |  |  | Time x Treatment | 6,66 | 0.820 | 0.558 |
|  | Timeout Rewards | Stim MRR / Stim MRR + DR | Treatment | 1,11 | 0.084 | 0.776 |
|  |  |  | Time | 6,66 | 0.217 | 0.969 |
|  |  |  | Time x Treatment | 6,66 | 1.206 | 0.314 |
|  |  | Inhib MRR / Inhib MRR + DR | Treatment | 1,11 | 0.263 | 0.618 |
|  |  |  | Time | 6,66 | 0.207 | 0.973 |
|  |  |  | Time x Treatment | 6,66 | 1.156 | 0.340 |
|  | Timeout Non-Rewards | Stim MRR / Stim MRR + DR | Treatment | 1,11 | 0.184 | 0.675 |
|  |  |  | Time | 6,66 | 1.293 | 0.272 |
|  |  |  | Time x Treatment | 6,66 | 1.547 | 0.176 |
|  |  | Inhib MRR / Inhib MRR + DR | Treatment | 1,11 | 0.834 | 0.380 |
|  |  |  | Time | 6,66 | 0.360 | 0.901 |
|  |  |  | Time x Treatment | 6,66 | 0.620 | 0.713 |

1. Active avoidance

| Phase | Parameters | Groups | Effect | df | F | p |
| --- | --- | --- | --- | --- | --- | --- |
| Learning | N# of EDST | Stim MRR / Stim MRR + DR | Treatment | 1,11 | 0.000 | 0.979 |
|  |  |  | Time | 6,66 | 22.516 | 0.000 |
|  |  |  | Time x Treatment | 6,66 | 0.903 | 0.497 |
|  |  | Inhib MRR / Inhib MRR + DR | Treatment | 1,11 | 0.238 | 0.642 |
|  |  |  | Time | 6,66 | 4.399 | 0.001 |
|  |  |  | Time x Treatment | 6,66 | 0.242 | 0.959 |
|  | N# of EDFS | Stim MRR / Stim MRR + DR | Treatment | 1,11 | 2.428 | 0.145 |
|  |  |  | Time | 6,66 | 9.510 | 0.000 |
|  |  |  | Time x Treatment | 6,66 | 2.806 | 0.016 |
|  |  | Inhib MRR / Inhib MRR + DR | Treatment | 1,11 | 0.605 | 0.465 |
|  |  |  | Time | 6,66 | 4.229 | 0.002 |
|  |  |  | Time x Treatment | 6,66 | 0.258 | 0.952 |
|  | N# of ESFL | Stim MRR / Stim MRR + DR | Treatment | 1,11 | 2.745 | 0.123 |
|  |  |  | Time | 6,66 | 2.295 | 0.043 |
|  |  |  | Time x Treatment | 6,66 | 1.056 | 0.396 |
|  |  | Inhib MRR / Inhib MRR + DR | Treatment | 1,11 | 0.843 | 0.393 |
|  |  |  | Time | 6,66 | 0.740 | 0.620 |
|  |  |  | Time x Treatment | 6,66 | 0.451 | 0.839 |
|  | Average latency | Stim MRR / Stim MRR + DR | Treatment | 1,13 | 0.380 | 0.548 |
|  |  |  | Time | 4,52 | 1.203 | 0.320 |
|  |  |  | Time x Treatment | 4,52 | 1.161 | 0.338 |
|  |  | Inhib MRR / Inhib MRR + DR | Treatment | 1,13 | 0.291 | 0.599 |
|  |  |  | Time | 4,52 | 0.969 | 0.434 |
|  |  |  | Time x Treatment | 4,52 | 0.475 | 0.753 |
| Reversal learning | N# of EDST | Stim MRR / Stim MRR + DR | Treatment | 1,13 | 0.002 | 0.958 |
|  |  |  | Time | 2,26 | 3.720 | 0.037 |
|  |  |  | Time x Treatment | 2,26 | 1.191 | 0.319 |
|  |  | Inhib MRR / Inhib MRR + DR | Treatment | 1,13 | 0.104 | 0.752 |
|  |  |  | Time | 2,26 | 0.544 | 0.587 |
|  |  |  | Time x Treatment | 2,26 | 0140 | 0.870 |
|  | N# of ESFL | Stim MRR / Stim MRR + DR | Treatment | 1,13 | 0.001 | 0.972 |
|  |  |  | Time | 2,26 | 4.061 | 0.029 |
|  |  |  | Time x Treatment | 2,26 | 1.374 | 0.270 |
|  |  | Inhib MRR / Inhib MRR + DR | Treatment | 1,13 | 0.104 | 0.752 |
|  |  |  | Time | 2,26 | 0.544 | 0.587 |
|  |  |  | Time x Treatment | 2,26 | 0.140 | 0.870 |

Abbreviations: CNO: clozapine-N-oxid; DR: dorsal raphe; EDST: Escape during stimulus; EDFS: Escape during footshock; ESFL: Escape failure; MRR: median raphe
