# Supplementary figures and images for: The role of the GABAergic cells of the median raphe region in reinforcement-based learning
Source: Sci Rep. 2024 Jan 12;14:1175. doi: 10.1038/s41598-024-51743-y (PMC10786920; doi:10.1038/s41598-024-51743-y)

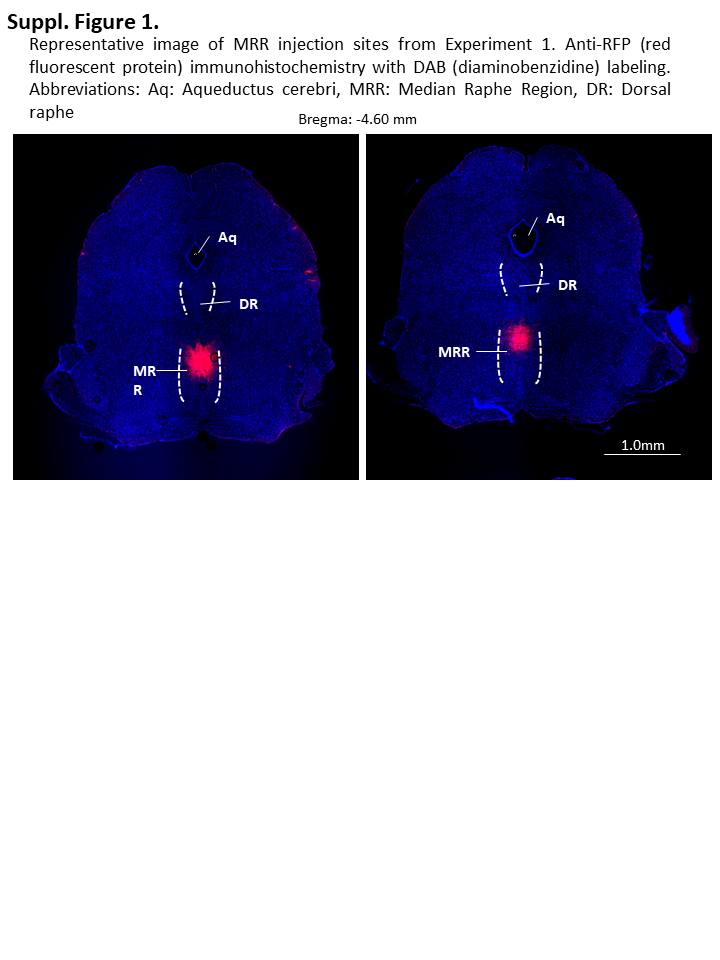

Supplement: Supplementary file 5 — Supplementary Figure 1. [file 41598_2024_51743_MOESM5_ESM.tif]

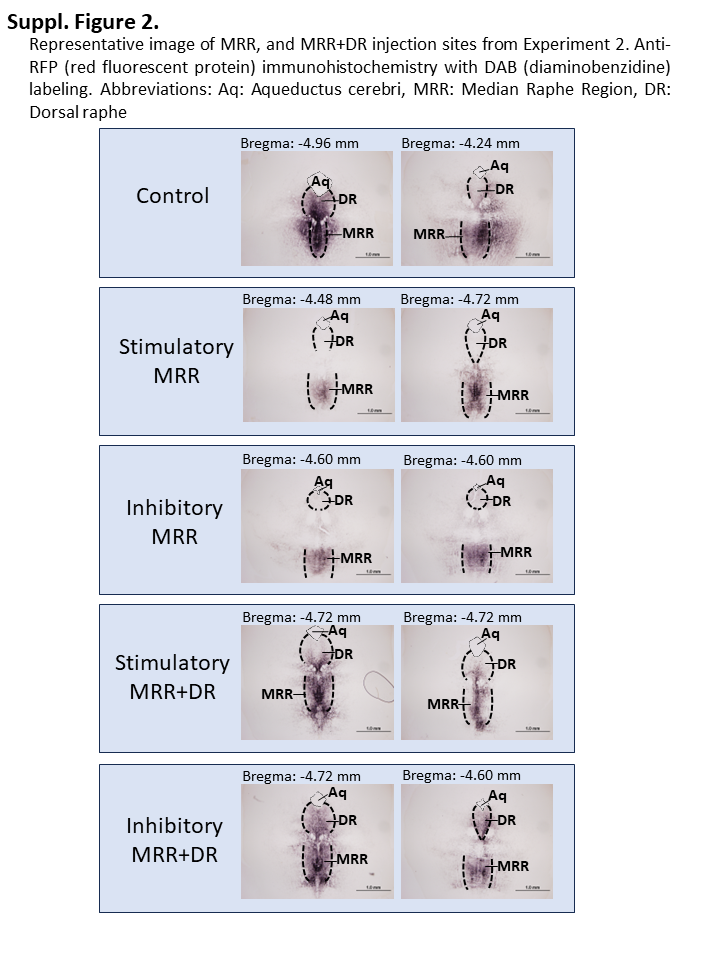

Supplement: Supplementary file 6 — Supplementary Figure 2. [file 41598_2024_51743_MOESM6_ESM.tif]
